# Primary Care Clinicians’ Prescribing Patterns of Reduced-Dose Direct Oral Anticoagulants for Extended-Phase Venous Thromboembolism Treatment

**DOI:** 10.3390/jcm13010096

**Published:** 2023-12-23

**Authors:** Danielle Groat, Karlyn A. Martin, Rachel P. Rosovsky, Kristen M. Sanfilippo, Manila Gaddh, Lisa Baumann Kreuziger, Elizabeth Federici, Scott C. Woller

**Affiliations:** 1 Center for Humanizing Critical Care, Intermountain Medical Center, Murray, UT 84107, USA; danielle.groat@imail.org; 2 Division of Hematology/Oncology, Department of Medicine, Northwestern University Feinberg School of Medicine, Chicago, IL 60611, USA; karlyn.martin@nm.org; 3 Division of Hematology/Oncology, Department of Medicine, Massachusetts General Hospital and Harvard Medical School, Boston, MA 02114, USA; rprosovsky@mgh.harvard.edu; 4 Division of Hematology, Department of Medicine, Washington University School of Medicine, St. Louis, MO 63110, USA; ksanfilippo@wustl.edu; 5 Department of Hematology and Medical Oncology, Emory University School of Medicine, Atlanta, GA 30307, USA; manila.gaddh@emory.edu; 6 Blood Research Institute, Versiti, Milwaukee, WI 53226, USA; lisakreuziger@versiti.org; 7 Penn State Hershey Medical Center, Hershey, PA 17033, USA; 8 Department of Medicine, Intermountain Medical Center, Murray UT 84107, USA; 9 Department of Internal Medicine, University of Utah School of Medicine, Salt Lake City, UT 84112, USA

**Keywords:** venous thromboembolism, direct oral anticoagulant, dose reduction, extended-phase treatment

## Abstract

The direct anticoagulants (DOACs), apixaban and rivaroxaban, are used for extended-phase treatment of venous thromboembolism (VTE) and have labeling for dose reduction for this indication. The objective of this study was to better understand primary care clinician prescribing patterns of apixaban and rivaroxaban for extended-phase anticoagulation. We conducted a 21-question survey targeting members of the American College of Physicians and United States Veterans Administration anticoagulation management services. Survey questions covered prescribing behaviors for dose reduction of apixaban and rivaroxaban for extended VTE treatment, as well as questions related to the respondent’s practice setting. We used logistic regression to assess associations between demographics and prescribing behaviors. We used k-means clustering to identify distinct groups of prescribing patterns. Among 227 respondents, most were attending physicians (60%) and one-third (34%) practiced in internal medicine or primary care. Most (59%) indicated they dose-reduced DOACs. Hospitalists (no outpatient care) were least likely to dose-reduce (OR 0.09 [95% CI 0.03–0.22]), as well as early-career clinicians (0.53 [0.30–0.91]). Pharmacists and clinicians who treat over 500 VTE patients annually were most likely to dose reduce (6.4 [2.9–16.3]), (2.9 [1.5–6.0]), respectively. We identified five clusters of dosing behaviors and characterized clinician makeup. Clusters were primarily differentiated by frequency of dose reduction, DOAC preference, and temporary re-escalation of doses. We identified clinician characteristics that are associated with dose-reduction prescribing behaviors; these analyses provide insight into where targeted interventions, such as protocolization and education, would be most beneficial.

## 1. Introduction

Anticoagulation is the treatment of choice for venous thromboembolism (VTE), including deep vein thrombosis (DVT) and pulmonary embolism (PE) [1,2,3,4]. Anticoagulation administration for a duration of 3–6 months is commonly elected for the initial treatment of acute VTE [1,2]. However, when the risk for recurrent VTE is assessed to be high, guideline recommendations advise to continue anticoagulant therapy beyond the initial treatment phase into “extended phase anticoagulation” [1,2]. Apixaban and Rivaroxaban are oral factor Xa inhibitors that are approved by the U.S. Food and Drug Administration for the treatment and secondary prevention of VTE [5,6,7,8,9]. Subsequent studies demonstrated that following an initial treatment phase of at least six months, reduced doses of apixaban (i.e., 2.5 mg twice daily) and rivaroxaban (i.e., 10 mg daily), when compared with initial treatment phase dose are equally safe and effective for the prevention of recurrent thrombosis and may be associated with a reduction in certain types of bleeding [8,10]. Dose-reduction studies have not been conducted with dabigatran or edoxaban [11]. However, in certain clinical situations such as in the case of VTE and cancer [12], obesity [13,14], or when new thrombosis occurs among patients taking a reduced dose of anticoagulation, patients are perceived as being at an especially high risk for recurrent thrombosis [15]. Limited evidence informs whether dose reduction is appropriate in these populations. The characteristics of clinicians that elect dose reduction compared with those who do not are also poorly understood.

Traditional survey analyses, where one question is analyzed with respect to another question, provide one-dimensional insight into specific aspects of the behaviors elicited by the survey. This methodology must also be driven by expert knowledge to ensure that appropriate questions are compared to generate meaningful insights. In contrast, without any prior knowledge of what types of groups may exist amongst survey respondents, incorporating all survey questions into an unsupervised machine learning analysis (i.e., clustering algorithm) can identify complex and multi-dimensional aspects of respondent behaviors, and this method has previously been used to describe prescribing behaviors in different settings [16]. The unlabeled data speak for themselves without bias from the investigators and can potentially lead to new, previously unidentified directions for future hypothesis testing.

In a former study, we reported the results of a survey that elicited physician preferences for dose reduction of apixaban and rivaroxaban for extended VTE treatment among attendees of an international thrombosis congress, and on Twitter feeds primarily associated with the congress [7]. Attending thrombosis specialists and primarily academic physicians were most represented in the publication. The objective of this study was to better understand primary care clinicians’ prescribing patterns for apixaban and rivaroxaban for extended-phase anticoagulation.

## 2. Methods

### 2.1. Survey Development

This work was conducted by members of the Venous ThromboEmbolism Network U.S. (VENUS) Subcommittee on Venous Thromboembolism Treatment and Anticoagulation Management. We made non-substantive changes to the survey we had previously developed [17] to account for a different targeted population of primary care clinicians including internal medicine physicians. We used REDCap to host the 21-question survey. The survey questions encompassed prescribing behaviors for dose reduction of apixaban and rivaroxaban for extended VTE treatment, e.g., frequency, risk factors, reescalation, and preference between medications. Additional questions were related to the respondent’s practice setting and geographic location.

### 2.2. Inclusion/Exclusion Criteria

A link to the survey was disseminated to target populations: the American College of Physicians (ACP), the U.S. Veterans Administration (VA) anticoagulation management services, and online via Twitter^TM^. The survey was made available from April of 2022 through January of 2023. A waiver of informed consent was granted by the Intermountain Health institutional review board (Reference No. 1051695). There were no exclusion criteria that made respondents ineligible for this study.

### 2.3. Statistical Analysis

Survey responses were presented with descriptive statistics. We grouped providers into mutually exclusive demographic groups based upon their profession (e.g., attending physician, pharmacist, midlevel, or other) and practice setting (e.g., inpatient, academic, outpatient or no expressly identified setting). Univariate logistic regression was used to assess associations between demographics and dose-reduction prescribing behavior. The independent variable was the demographic subgroup (while being compared to all other respondents not in that subgroup) and the dependent variable was whether or not they engaged in dose reduction of apixaban and rivaroxaban. Odds ratios greater than 1.0 indicate that the particular demographic subgroup engages in dose reduction more than their counterparts and is not to be interpreted as increasing the frequency (e.g., never [0%], rarely [between 0–25%], sometimes [25–50%], or usually [between 50–100%]) that one might dose reduce. Due to the exploratory nature of our analyses, we did not adjust for multiple comparisons. Statistical significance was set to 0.05.

### 2.4. Unsupervised Machine Learning

We used k-means clustering, an unsupervised machine learning algorithm [18], to discover prescribing patterns of apixaban and rivaroxaban amongst respondents. We first identified the optimal number of clusters (2 ≤ k ≤ 10) by maximizing the average silhouette score, a measure of cluster compactness and distinguishability [19], while maintaining adequate cluster size (*n* ≥ 25). We then visually inspected a scatterplot of the first two components of a principal component analysis to confirm adequate distinctness between the identified clusters. Dosing behavior frequencies and clinician demographics among the clusters were depicted with heatmaps.

All analyses were conducted with R version 4.0.3. We did not correct for multiple comparisons and consider all findings to be hypothesis generating.

## 3. Results

### 3.1. Demographics

There were 236 individuals who accessed the survey; 227 had a meaningful interaction with the survey, defined as respondents who answered at least two-thirds of the survey questions, and their responses were included in the analysis. Most (60%) were attending physicians, followed by pharmacists (24%). Approximately one-third were internal medicine specialists or primary care providers (34%), while 27% were medical subspecialists. Respondents from an academic hospital setting made up 44% of all respondents, while 31% were from the VA. The majority (53%) indicated that 80% or more of their time was dedicated to outpatient care. Representation with respect to years in practice (0 to ≥25) and number of patients seen (0 to ≥500) with DOAC prescriptions was evident across the cohort. (Table 1)

We identified 7 mutually exclusive demographic groups. The largest group with 53 (23%) respondents was comprised of pharmacists. The next largest group had 38 (17%) that self-identified as subspecialized attending physicians. The third group had 37 (16%) who provided no outpatient care and were largely hospitalists. Four additional groups were composed of attending primary care/internal medicine physicians in academic and not academic settings, mid-level providers, and there was a category for those that, in general, provide outpatient care. (Supplemental Table S1)

### 3.2. Prescribing Behaviors

The majority (59%) of respondents indicated that they dose reduce apixaban and rivaroxaban. Among those, 8.4% rarely do so (<25% of the time), 19% do sometimes (between 25–50% of the time), and 32% usually reduce (50–100% of the time). When asked in which patient populations they elect not to dose reduce, 82% said cancer, 74% recurrent VTE, and 70% prior VTE event on therapy. When clinicians were asked in which clinical situations they would elect to dose reduce, 79% said they would dose reduce for history of bleeding, 71% for distal DVT, and 63% for concurrent use of antiplatelet therapy. When asked if, upon electing dose reduction, they would consider re-escalation to a treatment dose, 40% indicated that they would do so. The most common reasons for temporary re-escalation to treatment dosing included cancer (32%) followed by surgery (30%). Most clinicians (77%) said they preferentially prescribe apixaban and 61% said they dose reduce both apixaban and rivaroxaban. (Table 2) Respondents identified 30% of the time that dosing frequency influenced the differential dose reduction of apixaban and rivaroxaban.

Of 35 respondents that were hospitalists, 86% said that they never dose reduce, whereas of 192 non-hospitalists, 67% said that they elect to dose reduce, with 38% usually doing so (50–100% of the time). Reasons for not dose reducing and diagnoses for dose reduction were all lower in the hospitalist group. Although non-hospitalists preferred prescribing apixaban (73%), hospitalists had an overwhelming preference for prescribing apixaban (94%). (Supplemental Table S2)

In univariate analysis of the seven groups (Table 3), clinicians who did not provide outpatient care, compared to all others who provided outpatient care, were least likely to dose reduce (odds ratio [OR] 0.09; 95% confidence interval [CI] 0.03–0.22; *p* < 0.001). Pharmacists had the highest odds of dose reduction (OR 6.4; 95% CI 2.9–16.3; *p* < 0.001), followed by subspecialized attending physicians (OR 2.6; 95% CI 1.2–6.0; *p* = 0.02). The remaining four groups, including attending physicians that were academic or not, mid-level providers, and respondents not otherwise categorized, did not statistically differ in their odds of dose reduction. Those with the fewest years in practice (<10) were less likely to dose reduce than their colleagues with more experience (OR 0.53; 95% CI 0.30–0.91; *p* = 0.02). The number of patients treated showed a pattern of lower odds of dose reduction with fewer patients and higher odds for those caring for larger volumes of patients on apixaban and rivaroxaban. Knowing whether a protocol was in place at their institution was indicative of prescribing dose reduction, while those who were unsure of any protocols were least likely to dose reduce (OR 0.22; 95% CI 0.11–0.43; *p* < 0.001).

### 3.3. Prescribing Behaviors Clusters

**Figure 1:**
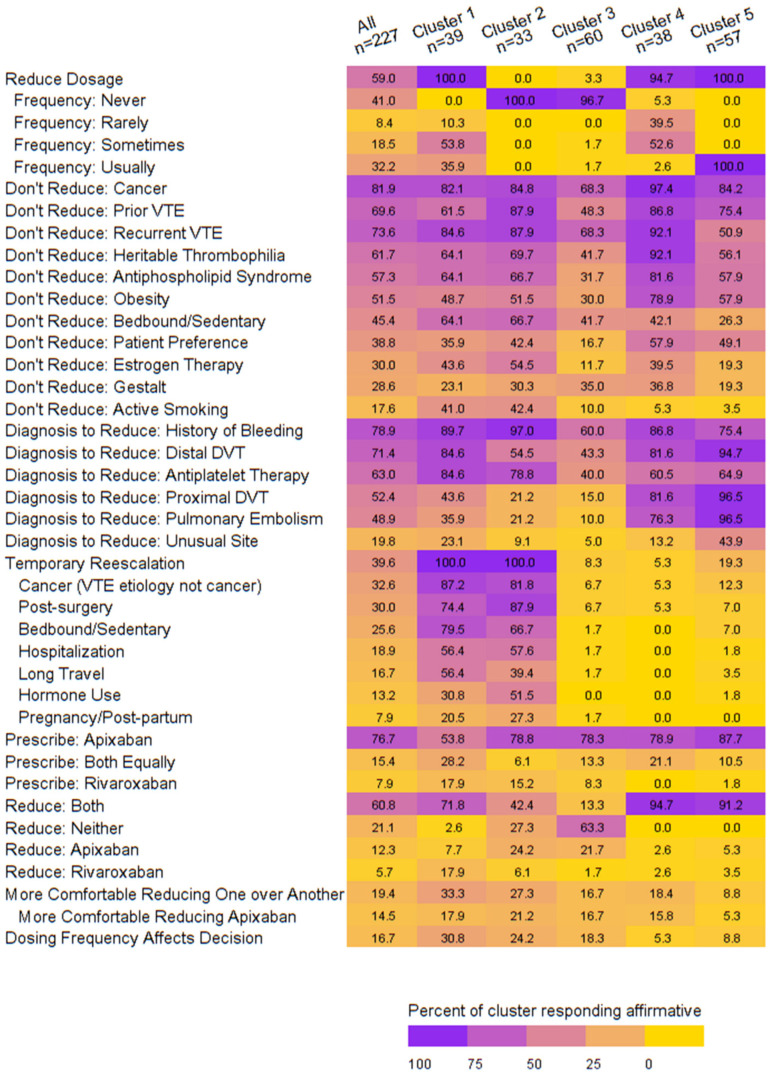
Heatmap of dosing behaviors by cluster.

We identified five clusters that demonstrated distinct patterns around dose reduction behaviors, as follows (Supplement Figure S1):

- Cluster 1: Sometimes or usually dose reduces, moderate rates for reasons/diagnoses to reduce/not reduce, always temporarily re-escalates dosing, prescribes both apixaban and rivaroxaban, reduces both, and is more comfortable reducing apixaban, possibly due to the dosing frequency.
- Cluster 2: Never dose reduces, moderate rates for reasons/diagnoses to reduce/not reduce, always temporarily re-escalates, preferentially prescribes apixaban, reduces both.
- Cluster 3: Never dose reduces, low rates for reasons/diagnoses to reduce/not reduce, does not temporarily escalate, preferentially prescribes apixaban, reduces neither.
- Cluster 4: Rarely or sometimes dose reduces, high rates for reasons to not dose reduce, does not temporarily re-escalate dosing, prescribes both with a preference for apixaban, reduces both.
- Cluster 5: Usually dose reduces, moderate rates for reasons to not reduce, high rates for diagnoses to reduce, infrequently temporarily re-escalates dosing, preferentially prescribes apixaban, reduces both. (Figure 1, Supplemental Table S3)

**Figure 2:**
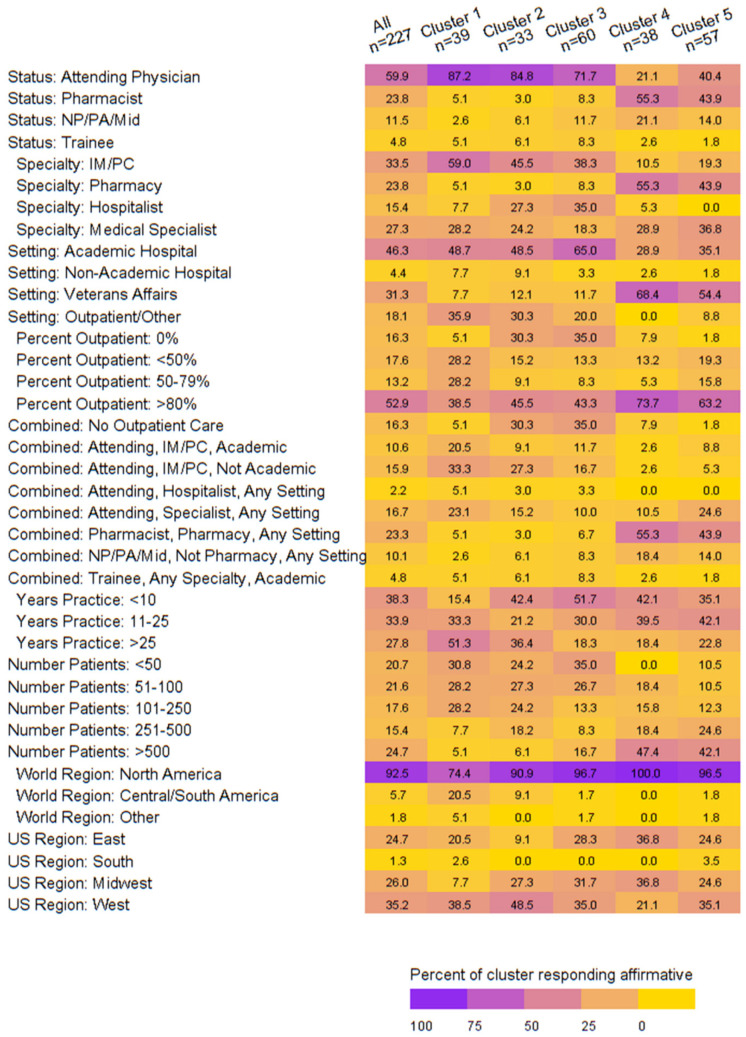
Heatmap of demographics by dosing behavior cluster.

When exploring the clinician demographics associated with the clusters of dose reduction behaviors, we noted the following:

- Cluster 1: Attending physicians in internal medicine/primary care treating patients in academic or outpatient settings outside of the United States (US) with >25 years in practice and <250 patients.
- Cluster 2: Attending physicians in academic/outpatient settings who do not provide outpatient care and treat <250 patients residing in the West and Midwest US.
- Cluster 3: Attending physicians or trainees or hospitalists who provide no outpatient care at an academic hospital with <25 years’ experience and <100 patients equally representing the East, West, and Midwest US.
- Cluster 4: Pharmacists at the VA with >80% of their time spent treating in the outpatient setting with <25 years’ experience treating >500 patients in the East or Midwest US.
- Cluster 5: Attendings with a medical specialty or pharmacists at the VA who spend >80% of their time in outpatient care with <25 years’ experience and >250 patients. (Figure 2)

## 4. Discussion

Here, we present the results of a survey that shows internal medicine physician and primary care clinician decision-making when considering dose reduction of apixaban and rivaroxaban for extended-phase treatment of VTE. While dose reduction for the prevention of VTE vitamin K antagonists (VKA) has been assessed, the low-intensity warfarin therapy compared with usual care intensity has been found to reduce efficacy without a reduction in bleeding risk [20]. For this reason, this practice has not been adopted. In comparison to VKAs, DOACs are unique. To the best of our knowledge, this is the first study to report apixaban and rivaroxaban dose-reduction prescribing behaviors in the primary care setting.

We were successful in ascertaining results from our target population in so far as attending physicians that provide outpatient care and pharmacists at the VA (with expertise and a dedicated role in anticoagulation management) constituted approximately two-thirds of the survey respondents. We found that most primary care clinicians elect to dose reduce for extended-phase anticoagulation. Pharmacists, medical specialists, and those prescribing apixaban and rivaroxaban in over 500 patients are most likely to dose reduce. Clustering of dosing behaviors emerged, and those clusters were primarily differentiated based on frequency of dose reduction and decision-making regarding the temporary re-escalation of apixaban or rivaroxaban dose. While electing dose reduction was dichotomously assessed as yes or no, we observed a nuanced approach to dose reduction frequency between clusters. For example, Cluster 1 elected dose reduction 100% of the time, and they did so ‘sometimes’ or ‘usually’. In comparison, Cluster 4 elected dose reduction 95% of the time yet did so ‘rarely’ or ‘sometimes’. Furthermore, we were able to characterize clinician demographics with each of the five dosing-behavior clusters. This finding is impactful because understanding the clustering of dosing behaviors and the demographic characteristics of those in each cluster has the potential to inform targeted educational campaigns surrounding dose reduction.

Our study builds on previous work by our group in which we administered the same survey where 87% of participants were attending physicians, 84% were at academic institutions, and 44% were thrombosis specialists, while the remaining 56% practiced in some other medical specialty [7]. The previous cohort of thrombosis specialists had higher rates of dose reduction and temporary re-escalation—83% and 62%, respectively—compared to our current cohort, where dose reduction and re-escalation were seen at rates of 59% and 40%; respectively. These studies provide important insight into how clinicians across different specialties and settings are practicing and prescribing DOACs. Our analyses provide insight into where targeted interventions, such as protocolization and education, would be most beneficial.

This study relied on a previously created survey to elicit apixaban and rivaroxaban prescribing behaviors, which is a strength of this work. We were able to identify prescribing patterns and identify associations with clinician characteristics. Perhaps unsurprisingly, we observed that internal medicine physicians that work as hospitalists almost never dose reduce. This observation is foreseeable, considering that the decision-making surrounding dose reduction is most likely to occur in an ambulatory setting when a patient is in a usual state of health. Likewise, it is much more likely that DOAC prescription by a hospitalist will be for an acute VTE event for which DOAC dose reduction consideration would not be indicated. Another study strength is the robust response to our survey by pharmacists at the VA. The VA has a well-established network of pharmacists who are dedicated to anticoagulation management and frequently deliver care under a collaborative practice agreement with the VA primary care physician. This process frequently leads to implementation of care processes models that are periodically revisited and inform best practice based on existing evidence and established guidelines, and we hypothesize that this practice design might be responsible for their predilection towards dose reduction.

A limitation of this work is that most respondents were from North America. The geographic uniformity of respondents may impact the generalizability of our findings, while the unanticipated responses provide additional insights we were not expecting. Also, as is frequently the case with self-report surveys, the results may suffer from recall or social desirability bias. The nature of the survey questions did not allow us to make any inferences with respect to clinical outcomes or other barriers clinicians may face when prescribing DOACs for VTE. In future work, we intend to combine this cohort with the cohort from our previous work to test and generate additional hypotheses related to dose-reduction prescribing behaviors. These findings will inform future interventions aimed at improving DOAC dose reduction practices for the right patients.

In conclusion, we observed that the majority of primary care clinicians elect to dose reduce apixaban and rivaroxaban for extended-phase anticoagulation therapy for the prevention of recurrent VTE. We identified clinician characteristics that are associated with dose-reduction prescribing behaviors. Further work is necessary to better understand potential factors across diverse clinicians, specialties, and settings to inform approaches that will lead to increased awareness and desirable DOAC prescribing behaviors.

## Figures and Tables

**Table 1:** Demographics.

Attribute	N (%)
Present status	
Attending Physician	136 (59.9%)
Pharmacist	54 (23.8%)
Nurse Practitioner/Physician Assistant/Mid-Level Provider (NP/PA/ML)	26 (11.5%)
Trainee	11 (4.8%)
Specialty	
Internal Medicine/Primary Care (IM/PC)	76 (33.5%)
Medical Specialist	62 (27.3%)
Pharmacy	54 (23.8%)
Hospitalist	35 (15.4%)
Setting	
Academic hospital	100 (44.1%)
Veterans Affairs (VA)	71 (31.3%)
Private practice	24 (10.6%)
Outpatient clinic	13 (5.7%)
Private non-teaching hospital	10 (4.4%)
Academic non-teaching hospital	5 (2.2%)
Other	4 (1.8%)
Percent of clinical time is outpatient care	
I provide no outpatient care	37 (16.3%)
1–50%	40 (17.6%)
50–79%	30 (13.2%)
≥80%	120 (52.9%)
Combined Status, Specialty, Setting, Outpatient Care	
Pharmacist, Pharmacy, Any Setting, Outpatient Care	53 (23.3%)
Attending Physician/Specialist, Any Setting, Outpatient Care	38 (16.7%)
Any Status, Any Specialty, Any Setting, No Outpatient Care	37 (16.3%)
Attending Physician, IM/PC, Not Academic/VA, Outpatient Care	36 (15.9%)
Attending Physician, IM/PC, Academic Hospital, Outpatient Care	24 (10.6%)
NP/PA/ML, Not Pharmacy, Any Setting, Outpatient Care	23 (10.1%)
Any Status, Not Pharmacy, Any Setting, Outpatient Care	16 (7.0%)
Years in practice	
≤10	87 (38.3%)
11–25	77 (33.9%)
≥25	63 (27.8%)
Number of patients prescribed DOACs annually	
≤50	47 (20.7%)
51–100	49 (21.6%)
101–250	40 (17.6%)
251–500	35 (15.4%)
≥500	56 (24.7%)
Protocol in place	
No	154 (67.8%)
Don’t Know	49 (21.6%)
Yes	24 (10.6%)
World Region	
North America	210 (92.5%)
Central/South America	13 (5.7%)
Other	4 (1.8%)
US Region	
West	80 (35.2%)
Midwest	59 (26.0%)
East	56 (24.7%)
South	3 (1.3%)

**Table 2:** DOAC dosing behaviors.

Attribute	N (%)
Reduce dosage	
Yes	134 (59.0%)
Frequency of dose reduction	
Never (0%)	93 (41.0%)
Rarely (between 0–25%)	19 (8.4%)
Sometimes (25–50% of the time)	42 (18.5%)
Usually (between 50–100%)	73 (32.2%)
Risk factors for no dose reduction	
Cancer	186 (81.9%)
Recurrent VTE *	167 (73.6%)
Prior VTE event or therapy	158 (69.6%)
Heritable Thrombophilia	140 (61.7%)
Antiphospholipid Syndrome	131 (56.7%)
Obesity, Body mass index > 30	117 (51.5%)
Bedbound/Immobility/Sedentary	103 (45.4%)
Patient Preference	88 (38.8%)
Estrogen-based hormone therapy	68 (30.0%)
Gestalt	65 (28.6%)
Active Smoking	40 (17.6%)
Insurance Coverage	18 (7.9%)
Age	15 (6.6%)
ECOG Performance Status	12 (5.3%)
Does not apply/Unaware	10 (4.4%)
Male Sex	4 (1.8%)
Diagnosis for reduction	
History of bleeding	179 (78.9%)
Distal DVT *	162 (71.4%)
Concurrent use of antiplatelet therapy	143 (63.0%)
Proximal DVT	119 (52.4%)
Pulmonary Embolism	111 (48.9%)
Unusual Site	45 (19.8%)
Temporary reescalation to therapeutic dose	
Yes	90 (39.6%)
Reason for temporary reescalation	
Cancer (if etiology for VTE was not cancer)	74 (32.6%)
Post-surgery	68 (30.0%)
Bedbound/Immobility/Sedentary	58 (25.6%)
Hospitalization	43 (18.9%)
Long travel	38 (16.7%)
Hormone use	30 (13.2%)
Pregnancy or post-partum	18 (7.9%)
DOAC prescribed most often	
Apixaban	174 (76.7%)
Prescribe Apixaban and Rivaroxaban equally	33 (14.5%)
Rivaroxaban	18 (7.9%)
Which medication dose-reduced	
Both	138 (60.8%)
Neither	47 (20.7%)
Apixaban	28 (12.3%)
Rivaroxaban	13 (5.7%)
More comfortable reducing one over another	
No	179 (78.9%)
Yes	44 (19.4%)
Which	
Apixaban	33 (14.5%)
Rivaroxaban	11 (4.8%)
Dose frequency affects decision	
Yes	38 (16.7%)

* VTE: venous thromboembolism; DVT: deep vein thrombosis.

**Table 3:** Exploratory analysis of associations between dose reduction behavior and demographics. Odds ratios were estimated by comparing each demographic group with all other survey respondents.

Demographic	Odds Ratio (95% CI)	*p*-Value
Combined Status, Specialty, and Setting		
No Outpatient Care	0.094 (0.034–0.222)	<0.001
Attending Physician, IM/PC, Academic Hospital	0.968 (0.413–2.345)	0.941
Attending Physician, IM/PC, Not Academic/VA	0.566 (0.274–1.159)	0.119
Attending Physician, Specialist, Any Setting	2.578 (1.198–6.049)	0.021
Pharmacist, Pharmacy, Any Setting	6.422 (2.911–16.28)	<0.001
NP/PA/Mid-Level, Not Pharmacy, Any Setting	1.666 (0.679–4.495)	0.282
Any Status, Not Pharmacy, Any Setting	0.389 (0.128–1.088)	0.078
Years in practice		
<10	0.526 (0.304–0.906)	0.021
11–25	1.725 (0.977–3.098)	0.063
>25	1.180 (0.654–2.160)	0.585
Number of patients where you are involved in DOAC prescriptions		
<50	0.384 (0.196–0.735)	0.004
51–100	0.659 (0.348–1.249)	0.199
101–250	1.050 (0.527–2.139)	0.891
251–500	1.399 (0.668–3.059)	0.383
>500	2.908 (1.494–5.985)	0.002
Protocol in place		
No	2.310 (1.312–4.100)	0.004
Don’t Know	0.219 (0.108–0.426)	<0.001
Yes	2.250 (0.901–6.419)	0.099

## Data Availability

The study protocol approved by Intermountain Health institutional review board (IRB) only permits aggregate survey data to be shared outside the institution. Requests for access to survey data require a protocol amendment and subsequent approval from Intermountain’s IRB.

## Supplementary Materials

The following supporting information can be downloaded at: https://www.mdpi.com/article/10.3390/jcm13010096/s1, Figure S1: Visual inspection of first two principal components suggests good separation between the five identified dosing behavior clusters.; Table S1: Demographic details of combined groups; Table S2: Comparison of dosing strategies of non-hospitalists and hospitalists, reported as n (%); Table S3: Dosing behaviors and demographics by cluster.
