# Supplementary material for: Primary Care Clinicians’ Prescribing Patterns of Reduced-Dose Direct Oral Anticoagulants for Extended-Phase Venous Thromboembolism Treatment
Source: J Clin Med. 2023 Dec 23;13(1):96. doi: 10.3390/jcm13010096 (PMC10779925; doi:10.3390/jcm13010096)
Supplement: Supplementary file 1 [file jcm-13-00096-s001.zip › jcm-2776617-supplementary.pdf]

ONLINE SUPPLEMENT

Primary Care Clinicians' Prescribing Patterns of Reduced-Dose Direct Oral Anticoagulants for Extended-Phase Venous Thromboembolism Treatment

Danielle Groat, PhD, Karlyn A. Martin MD, MS, Rachel P. Rosovsky MD, MPH, Kristen M. Sanfilippo MD, Manila Gaddh MD, Lisa Baumann Kreuziger MD, MS, Elizabeth Federici MD, Scott C. Woller MD for the Venous thromboEmbolism Network US (VENUS) VTE Treatment and Anticoagulation Management Group

Supplementary Table S1: Demographic details of combined groups.

Combined Group	Frequency	Present Status				Specialty				Setting					Outpatient Time			
		Attending Physician	Pharmacist	NP/PA/Mid=Level	Trainee	IM/PC	Medical Specialist	Pharmacy	Hospitalist	Academic	VA	Private	Outpatient	Other	None	< 50%	50-79%	> 80%
Pharmacist, Pharmacy, Any Setting, Outpatient	53	0	53	0	0	0	0	53	0	2	51	0	0	0	0	3	2	48
Attending Physician, Medical Specialist, Any Setting, Outpatient	38	38	0	0	0	0	38	0	0	29	2	6	1	0	0	12	15	11
Any Status, Specialty, or Setting, No outpatient	37	33	1	3	0	4	2	1	30	34	0	3	0	0	37	0	0	0
Attending Physician, IM/PC, Not Academic/VA, Outpatient	36	36	0	0	0	36	0	0	0	0	0	24	8	4	0	3	5	28

Attending Physician, IM/PC, Academic, Outpatient	24	24	0	0	0	24	0	0	0	24	0	0	0	0	0	12	2	10
NA/PA/Mid, Not Pharmacy, Any Setting, Outpatient	23	0	0	23	0	9	14	0	0	2	18	0	3	0	0	3	0	20
Any Status, Not Pharmacy, Any Setting, Outpatient	16	5	0	0	11	3	8	0	5	14	0	1	1	0	0	7	6	3

Supplementary Table S2: Comparison of dosing strategies of non-hospitalists and hospitalists, reported as n (%).

Attribute	Hospitalist, n = 35	Not Hospitalist. n=192
Reduce dosage		
Yes	5 (14.3%)	129 (67.2%)
Frequency of dose reduction		
Never (0%)	30 (85.7%)	63 (32.8%)
Rarely (between 0-25%)	3 (8.6%)	16 (8.3%)
Sometimes (25-50% of the time)	1 (2.9%)	41 (21.4%)
Usually (between 50-100%)	1 (2.9%)	72 (37.5%)
Risk factors for no dose reduction		
Cancer	25 (71.4%)	161 (83.9%)
Recurrent VTE	25 (71.4%)	142 (74.0%)
Prior VTE event or therapy	21 (60.0%)	137 (71.4%)
Heritable Thrombophilia	17 (48.6%)	123 (64.1%)
Antiphospholipid Syndrome	19 (54.3%)	111 (57.8%)
Obesity (BMI>30)	8 (22.9%)	109 (56.8%)
Bedbound/Immobility/Sedentary	18 (51.4%)	85 (44.3%)
Patient Preference	7 (20.0%)	81 (42.2%)
Estrogen-based hormone therapy	1 (2.9%)	17 (8.9%)
Gestalt	8 (22.9%)	60 (31.2%)
Active Smoking	2 (5.7%)	10 (5.2%)
Insurance Coverage	5 (14.3%)	35 (18.2%)
Age	2 (5.7%)	13 (6.8%)
ECOG Performance Status	11 (31.4%)	54 (28.1%)
Does not apply/Unaware	7 (20.0%)	3 (1.6%)
Male Sex	0 (0%)	4 (2.1%)
Diagnosis for reduction		
History of bleeding	22 (62.9%)	157 (81.8%)
Distal DVT	18 (51.4%)	144 (75.0%)
Concurrent use of antiplatelet therapy	16 (45.7%)	127 (66.1%)
Proximal DVT	8 (22.9%)	111 (57.8%)

Pulmonary Embolism	8 (22.9%)	103 (53.6%)
Unusual Site	1 (2.9%)	44 (22.9%)
Temporary reescalation		
Yes	16 (45.7%)	74 (38.5%)
Reason for temporary reescalation to therapeutic dose		
Cancer (if etiology for VTE was not cancer)	14 (40.0%)	60 (31.2%)
Post-surgery	12 (34.3%)	56 (29.2%)
Bedbound/Immobility/Sedentary	9 (25.7%)	49 (25.5%)
Hospitalization	6 (17.1%)	37 (19.3%)
Long travel	4 (11.4%)	34 (17.7%)
Hormone use	6 (17.1%)	24 (12.5%)
Pregnancy or post-partum	1 (2.9%)	17 (8.9%)
DOAC prescribed most often		
Apixaban	33 (94.3%)	141 (73.4%)
Prescribe Apixaban and Rivaroxaban Equally	2 (5.7%)	31 (16.1%)
Rivaroxaban	0 (0%)	18 (9.4%)
Which medication dose-reduced		
Both	8 (22.9%)	130 (67.7%)
Neither	14 (40.0%)	33 (17.2%)
Apixaban	13 (37.1%)	15 (7.8%)
Rivaroxaban	0 (0%)	13 (6.8%)
More comfortable reducing one over another		
Yes	12 (34.3%)	32 (16.7%)
Which		
Apixaban	12 (34.3%)	21 (10.9%)
Rivaroxaban	0 (0%)	11 (5.7%)
Dosing frequency affects decision		
Yes	6 (17.1%)	32 (16.7%)

Supplementary Table S3: Dosing behaviors and demographics by cluster.

Cluster	Dosing Behaviors	Demographics
1	Sometimes or usually dose-reduces, moderate rates for reasons/diagnoses to reduce/not reduce, always temporarily re-escalates dosing, prescribes both apixaban and rivaroxaban, reduces both, and is more comfortable reducing apixaban possibly due to the dosing frequency.	Attending physicians in internal medicine/primary care treating patients in academic or outpatient settings outside of the United States (US) with >25 years in practice and <250 patients.
2	Never dose-reduces, moderate rates for reasons/diagnoses to reduce/not reduce, always temporarily re-escalates, preferentially prescribes apixaban, reduces both.	Attending physicians in academic/outpatient settings who do not provide outpatient care and treat <250 patients residing in the West and Midwest US.
3	Never dose-reduces, low rates for reasons/diagnoses to reduce/not reduce, does not temporarily escalate, preferentially prescribes apixaban, reduces neither.	Attending physicians or trainees or hospitalist who provide no outpatient care at an academic hospital with <25 years' experience and <100 patients equally representing the East, West and Midwest US.

4	Rarely or sometimes dose-reduces, high rates for reasons to not dose-reduce, does not temporarily re-escalate dosing, prescribes both with a preference for apixaban, reduces both.	Pharmacists at the VA with >80% of their time for treating in the outpatient setting with <25 years' experience treating >500 patients in the East or Midwest US.
5	Usually dose-reduces, moderate rates for reasons to not reduce, high rates for diagnoses to reduce, infrequently temporarily re-escalates dosing, preferentially prescribes apixaban, reduces both.	Attendings with a medical specialty or pharmacists at the VA who provide >80% of their time in outpatient care with <25 years' experience and >250 patients.

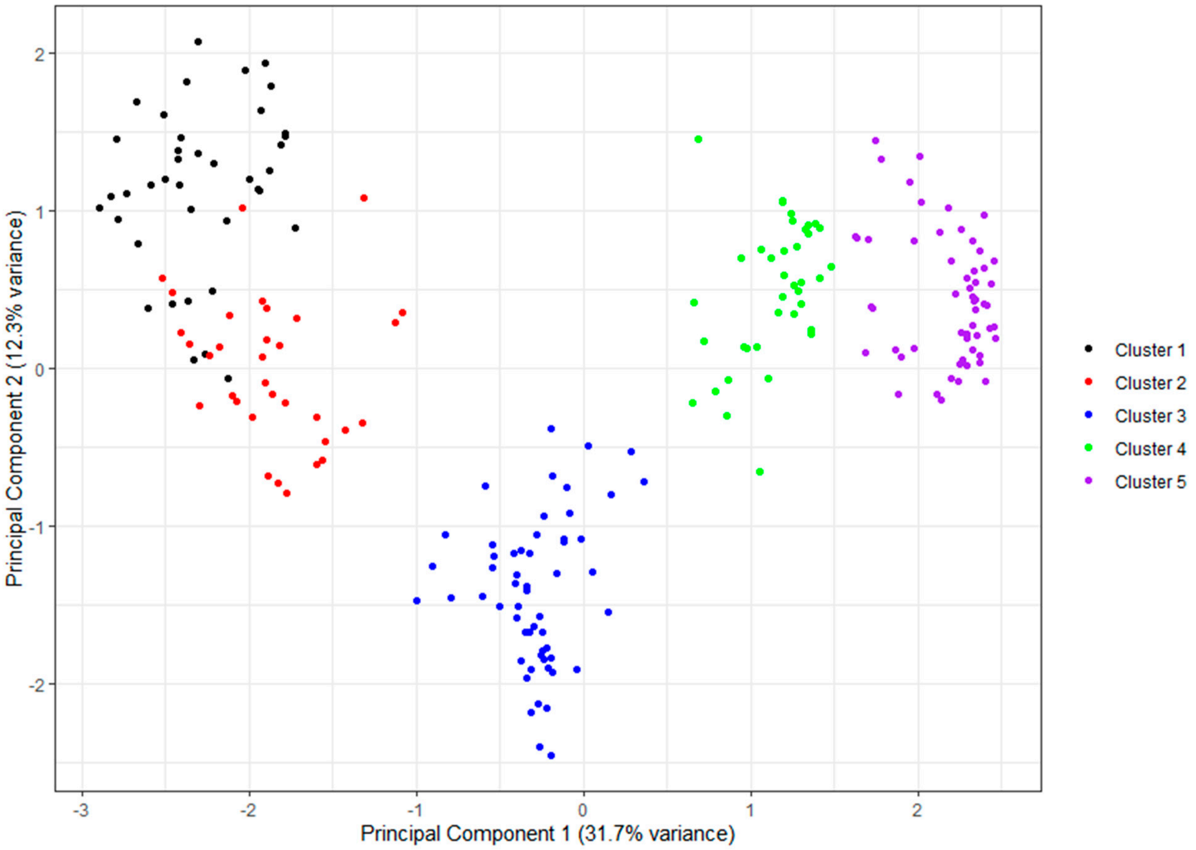

Supplementary Figure S1: Visual inspection of first two principal components suggests good separation between the five identified dosing behavior clusters.
